# Third-party governance and artificial intelligence for diabetes management in primary health care, China

**DOI:** 10.2471/BLT.25.295347

**Published:** 2026-05-27

**Authors:** Wenxi Tang, Qian Peng, Minlin Li, Na Zheng, Tao Zhang, Yipeng Guo, Xing Lin Feng

**Affiliations:** a Department of Public Administration, China Pharmaceutical University, Nanjing, China.; b Tianjin Medical Insurance Bureau, Tianjin, China.; c Tianjin Health Commission, Tianjin, China.; d Department of Health Policy and Management, Peking University School of Public Health, 38 Xueyuan Road, Beijing, 100191, China.

## Abstract

**Problem:**

Fragmented primary health care in China fails to tackle the growing burden of noncommunicable diseases. Despite substantial investment, fewer than half of patients with diabetes achieve glycaemic control.

**Approach:**

Tianjin’s 2020–2023 reform established a public–private partnership model where WeDoctor managed community health centres under a capitation scheme. This strategy integrated: (i) monthly prepaid capitation covering all diabetes-related outpatient services; (ii) claims auditing and clinical decision support using artificial intelligence (AI); (iii) dedicated health managers for care coordination; and (iv) redesigned services incorporating complication screening and digital medication management. A pilot study including 494 945 patients with diabetes compared three care models from 2022 to 2023: WeDoctor–community health centre care, hospital care and usual care.

**Local setting:**

Tianjin city serves 15 million residents through 177 hospitals and 266 community health centres. Chronic disease management is fragmented: the Health Commission regulates care standards, while the Insurance Bureau controls funding.

**Relevant changes:**

Visits for diabetes at WeDoctor health centres increased 2.6% (0.7/26.6) but declined 10.6% (−3.8/35.7) for hospital-based care and 2.3% (−0.8/34.1) for usual care. All groups reduced outpatient expenditure. The WeDoctor model generated a 37.62 million United States dollars (US$) surplus, boosted health centre diabetes revenue by 65% (US$ 154 577/237 805) and raised physician annual salaries by 30% (US$ 5172/17 241). More than three quarters of patients expressed satisfaction with and trust in the WeDoctor model.

**Lessons learnt:**

Integrating capitation financing with third-party governance and AI support can strengthen primary health care, contain costs and enhance patient-centred care.

## Résumé

**Problème:**

La fragmentation des soins de santé primaires en Chine ne permet pas de faire face au fardeau croissant des maladies non transmissibles. Malgré des investissements considérables, moins de la moitié des patients diabétiques parviennent à contrôler leur glycémie.

**Approche:**

La réforme de Tianjin pour la période 2020–2023 a mis en place un modèle de partenariat public-privé dans le cadre duquel WeDoctor gérait les centres de santé communautaires selon un système de forfait par habitant. Cette stratégie intégrait: (i) un forfait mensuel prépayé couvrant tous les services de consultation externe liés au diabète; (ii) un audit des demandes de remboursement et un soutien aux décisions cliniques à l’aide de l’intelligence artificielle (IA); (iii) des cadres de santé dédiés à la coordination des soins; et (iv) des services repensés pour intégrer le dépistage des complications et la gestion numérique des médicaments. Une étude pilote portant sur 494 945 patients diabétiques a comparé trois modèles de soins entre 2022 et 2023: les soins dispensés par WeDoctor et les centres de santé communautaires, les soins hospitaliers et les soins habituels.

**Environnement local:**

La ville de Tianjin dessert 15 millions d’habitants grâce à 177 hôpitaux et 266 centres de santé communautaires. La prise en charge des maladies chroniques est fragmentée: la Commission de la santé réglemente les normes de soins, tandis que le Bureau des assurances oriente le financement.

**Changements significatifs:**

Les consultations pour le diabète dans les centres de santé WeDoctor ont augmenté de 2,6% (0,7/26,6), mais ont diminué de 10,6% (−3,8/35,7) pour les soins hospitaliers et de 2,3% (−0,8/34,1) pour les soins habituels. Tous les groupes ont réduit leurs dépenses pour des soins ambulatoires. Le modèle WeDoctor a généré un excédent de 37,62 millions de dollars américains (USD), augmenté les recettes des centres de santé liées au diabète de 65% (154 577 / 237 805 USD) et accru de 30% les salaires annuels des médecins (5 172 / 17 241 USD). Plus des trois quarts des patients ont exprimé leur satisfaction et leur confiance dans le modèle WeDoctor.

**Leçons tirées:**

L’intégration du financement par forfait à une gouvernance par des tiers et à un soutien par l’IA permet de renforcer les soins de santé primaires, de maîtriser les coûts et d’améliorer les soins axés sur le patient.

## Resumen

**Situación:**

La fragmentación de la atención primaria de salud en China no logra hacer frente a la creciente carga de las enfermedades no transmisibles. A pesar de las importantes inversiones realizadas, menos de la mitad de los pacientes con diabetes logran un control glucémico adecuado.

**Enfoque:**

La reforma aplicada en Tianjin entre 2020 y 2023 estableció un modelo de asociación público-privada en el que WeDoctor gestionaba centros comunitarios de salud mediante un sistema de capitación. Esta estrategia integró: (i) una capitación mensual prepagada que cubría todos los servicios ambulatorios relacionados con la diabetes; (ii) la auditoría de reclamaciones y el apoyo a la toma de decisiones clínicas mediante inteligencia artificial (IA); (iii) gestores sanitarios dedicados a la coordinación asistencial; y (iv) servicios rediseñados que incorporaban el cribado de complicaciones y la gestión digital de la medicación. Un estudio piloto que incluyó a 494 945 pacientes con diabetes comparó tres modelos asistenciales entre 2022 y 2023: la atención prestada por WeDoctor en centros comunitarios de salud, la atención hospitalaria y la atención habitual.

**Marco regional:**

La ciudad de Tianjin presta servicios a 15 millones de habitantes a través de 177 hospitales y 266 centros comunitarios de salud. La gestión de las enfermedades crónicas está fragmentada: la Comisión de Salud regula los estándares asistenciales, mientras que la Oficina de Seguros controla la financiación.

**Cambios importantes:**

Las consultas por diabetes en los centros de salud de WeDoctor aumentaron un 2,6% (0,7/26,6), mientras que disminuyeron un 10,6% (-3,8/35,7) en la atención hospitalaria y un 2,3% (-0,8/34,1) en la atención habitual. Todos los grupos redujeron el gasto ambulatorio. El modelo WeDoctor generó un superávit de US$ 37,62 millones, aumentó en un 65% los ingresos por atención de la diabetes en los centros de salud (US$ 154 577/237 805) e incrementó en un 30% los salarios anuales de los médicos (US$ 5172/17 241). Más de tres cuartas partes de los pacientes expresaron satisfacción y confianza en el modelo WeDoctor.

**Lecciones aprendidas:**

La integración de la financiación mediante capitación con la gobernanza por terceros y el apoyo de la IA puede fortalecer la atención primaria de salud, contener los costes y mejorar la atención centrada en el paciente.

## ملخص

**المشكلة:**

تعجز الرعاية الصحية الأولية المجزأة في الصين عن مواجهة العبء المتزايد الناتج عن الأمراض غير المعدية. والرغم من الاستثمارات الضخمة، فإن أقل من نصف مرضى السكري يحققون السيطرة على مستوى السكر في الدم.

**الأسلوب:**

أدت الإصلاحات في مدينة تيانجين خلال الفترة من 2020 إلى 2023 لتأسيس نموذج للشراكة بين القطاعين العام والخاص، حيث تولت شركة WeDoctor إدارة المراكز الصحية المجتمعية ضمن نظام الدفع الفردي. وقد اشتملت هذه الاستراتيجية على ما يلي: (1) دفع شهري مسبق يغطي جميع خدمات العيادات الخارجية المتعلقة بمرض السكري؛ و(2) تدقيق المطالبات ودعم القرارات الإكلينيكية باستخدام الذكاء الاصطناعي؛ و(3) مديرين للرعاية الصحية متخصصين لتنسيق الرعاية؛ و(4) خدمات أُعيد تنسيقها لتشمل فحص المضاعفات والإدارة الرقمية للأدوية. وقد قارنت دراسة تجريبية، شملت 494945 مريضًا بمرض السكري، بين ثلاثة نماذج للرعاية خلال الفترة من 2022 إلى 2023: الرعاية المقدمة في مراكز WeDoctor الصحية المجتمعية، والرعاية في المستشفيات، والرعاية المعتادة.

**المواقع المحلية:**

تخدم مدينة تيانجين 15 مليون نسمة من خلال 177 مستشفى و266 مركزًا صحيًا مجتمعيًا. يتسم نظام إدارة الأمراض المزمنة بأنها مجزأة، حيث تتولى لجنة الصحة تنظيم معايير الرعاية، بينما يتحكم مكتب التأمين في التمويل.

**التغيّرات ذات الصلة:**

ارتفعت زيارات مرضى السكري إلى مراكز WeDoctor الصحية بنسبة %2.6 (0.7/26.6)، بينما انخفضت بنسبة %10.6 (3.8-/35.7) للرعاية المقدمة في المستشفيات، وبنسبة %2.3 (0.8-/34.1) للرعاية المعتادة. وقد خفضت جميع المجموعات نفقات العيادات الخارجية. وحقق نموذج WeDoctor فائضًا قدره 37.62 مليون دولار أمريكي، ورفع عائد خدمات مرض السكري في مراكز الرعاية الصحية بنسبة %65 (154577/237805 دولارًا أمريكيًا)، ورفع الرواتب السنوية للأطباء بنسبة %30 (5172/17241 دولارًا أمريكيًا). وأعرب أكثر من ثلاثة أرباع المرضى عن رضاهم وثقتهم بنموذج WeDoctor.

**الدروس المستفادة:**

إن دمج تمويل الدفع الفردي مع حوكمة الجهة الخارجية، ودعم الذكاء الاصطناعي، يمكن أن يعزز الرعاية الصحية الأولية، ويحد من التكاليف، ويعزز الرعاية التي تركز على المريض.

## 摘要

**问题:**

中国基层医疗卫生比较分散，难以应对非传染性疾病日益严峻的负担。尽管投入较大，仍仅有不到半数的糖尿病患者实现血糖达标。

**方法:**

天津市 2020-2023 年基层医改推行公私协同模式，由微医采用“按人头付费”制度协助管理社区卫生服务中心。该举措涵盖以下四点：(1) 按月预付的人头付费，覆盖与糖尿病相关的所有门诊服务；(2) 采用人工智能来开展医保费用审核和临床决策支持；(3) 配备专职的健康管理人员负责照护协调；以及 (4) 重构服务内容，新增并发症筛查并纳入数字化用药管理。一项于 2022 年至 2023 年开展的纳入 494945 名糖尿病患者的试点研究，对比了三种诊疗模式：微医联合社区卫生服务中心模式、医院诊疗模式及常规诊疗模式。

**当地状况:**

天津市现有 177 家医院和 266 家社区卫生服务中心，服务 1500 万居民。慢性病管理体系分散：卫生健康部门监管诊疗服务标准，医保部门负责管控经费。

**相关变化:**

微医联合卫生服务中心模式下的糖尿病就诊人次增长 2.6% (0.7/26.6)；而医院诊疗模式下的就诊人次下降 10.6% (−3.8/35.7)，常规诊疗模式下的就诊人次下降 2.3% (−0.8/34.1)。所有模式下的门诊费用均有所减少。微医模式产生 3762 万美元的结余，卫生服务中心的糖尿病相关收入提升 65%（154577/237805 美元），医生年薪提高 30%（5172/17241 美元）。超过四分之三的患者对微医模式表示满意和信任。

**经验教训:**

按人头付费模式与第三方治理及人工智能技术相结合，能够夯实基层医疗卫生服务能力、控制医疗成本，同时提升以患者为中心的服务质量。

## Резюме

**Проблема:**

Фрагментированная структура первичного медико-санитарного обслуживания в Китае не справляется с растущим бременем неинфекционных заболеваний. Несмотря на существенные инвестиции, гликемического контроля удается достичь менее чем для половины пациентов с диабетом.

**Подход:**

В рамках реформы, проведенной в Тяньцзине в 2020–2023 годах, была создана модель государственно-частного партнерства, где компания WeDoctor управляла муниципальными центрами здравоохранения по схеме подушевого финансирования. Стратегия включала: (i) ежемесячное предварительное подушевое финансирование, которое покрывало все амбулаторные услуги, связанные с диабетом; (ii) аудит страховых требований и помощь в принятии клинических решений с использованием искусственного интеллекта (ИИ); (iii) назначение специальных координаторов медицинского обслуживания; (iv) изменения в структуре обслуживания, в том числе скрининг осложнений и цифровое управление медикаментозной терапией. В пилотном исследовании с участием 494 945 пациентов с диабетом сравнивались три модели лечения в период с 2022 по 2023 год: помощь на базе муниципальных центров здравоохранения WeDoctor, лечение в больнице и обычное медицинское обслуживание.

**Местные условия:**

В городе Тяньцзинь на 15 миллионов жителей приходится 177 больниц и 266 муниципальных центров здравоохранения. Ведение пациентов с хроническими заболеваниями осуществляется фрагментированно: Комиссия по здравоохранению регулирует стандарты оказания помощи, а Бюро страхования контролирует финансирование.

**Осуществленные перемены:**

Частота посещения медицинских центров компании WeDoctor по поводу диабета возросла на 2,6% (+0,7 при исходных 26,6), однако снизилась на 10,6% (–3,8 при исходных 35,7) для лечения на базе стационара и на 2,3% (–0,8 при исходных 34,1) для обычного медицинского обслуживания. Во всех группах сократились издержки на амбулаторное лечение. Модель WeDoctor обеспечила профицит в размере 37,62 млн долл. США, увеличив доходы медицинских центров от оказания помощи пациентам с диабетом на 65% (+154 577 долл. США при исходных 237 805), а также повысив ежегодную зарплату врачей на 30% (+5172 долл. США при исходных 17 241). Более трех четвертей пациентов выразили удовлетворенность моделью WeDoctor и доверие к ней.

**Выводы:**

Сочетание подушевого финансирования, модели управления силами сторонних организаций и использования искусственного интеллекта может укрепить систему первичной медико-санитарной помощи, оптимизировать издержки и повысить ориентированность медицинской помощи на потребности пациентов.

## Introduction

Primary health care in low- and middle-income countries is under-resourced and fragmented, failing to effectively manage the rising burden of noncommunicable diseases. This fragmentation is acute in China, where rapid epidemiological transitions have outpaced health-system reforms. China faces a diabetes crisis: the prevalence is estimated at about 12.4% but only about a half of cases achieve glycaemic control.^1^ Although primary care facilities are the first point of contact, they are underused, indicating a structural failure rather than lack of resources.

While China has increased investment from 2.8 billion United States dollars (US$) in 2008 to US$ 33.7 billion in 2019 and established the National Essential Public Health Service Programme,^2^ the capacity of primary care networks to manage noncommunicable diseases has decreased. The main failure lies in the misalignment of financing and service delivery. The absence of a mandatory referral system allows patients unrestricted access to hospital outpatient care, which undermines the role of primary care. Furthermore, the government funds preventive services at primary care, while social health insurance funds curative services, creating perverse incentives that encourage service fragmentation and volume-driven care rather than good health outcomes.^3^

To guide patients to seek care at the primary level and restructure the health-care delivery system, policy-makers have implemented top-down interventions, including family doctor contracting, tiered medical treatment and different reimbursement rates. However, these policies have been largely ineffective.^4^ This failure reflects a disconnect between the macro-level policy vision of patient-centred integrated care and the micro-level institutional reality characterized by fragmentation of preventive and curative financing and competition rather than cooperation between primary care facilities and hospitals.

Here, we describe a system-wide reform in Tianjin that piloted two innovations for diabetes care: a capitation payment model and a private third-party organizer. We analyse first-year outcomes (2022–2023) to inform future scaling up and optimization, drawing on policy reviews, semi-structured interviews with health-system administrators and frontline providers, and de-identified electronic health records.

## Local setting

Tianjin city serves 15 million residents through 177 hospitals and 266 community health centres. The Health Commission regulates care standards, while the Insurance Bureau controls funding. Chronic disease management is fragmented: health centres receive US$ 14 a year per capita for preventive management of chronic diseases (e.g. lifestyle counselling and diabetes and hypertension monitoring), excluding clinical treatment. In contrast, insurance separately reimburses curative services. Patients can choose providers, exacerbating care misalignment. 

The government partners with WeDoctor, a private health management company. In Tianjin, WeDoctor maintains a multidisciplinary workforce of 300–400 personnel, including semi-professional health managers, liaison managers and a technical and administrative support team. Health managers hold a National Health Manager Certification and provide comprehensive health management for patients using artificial intelligence (AI)-enabled tools (Box 1).

Box 1 **Recruitment, training, and compensation structure of health managers, pilot on diabetes management in primary health care, China**

***Recruitment pipeline***

The health manager workforce at WeDoctor Tianjin comprises two primary recruitment channels: approximately 40% are graduates from WeDoctor's proprietary Hainan Health Management College (annual intake: 300 students); remaining 60% are locally recruited within Tianjin's health care sector.

All personnel hold the National Health Manager Certification issued by institutions designated by the Chinese Ministry of Human Resources and Social Security, and 74% additionally possess clinical qualifications, such as nursing licenses or physician credentials.

***Training protocol***

The training system includes: probationary phase (at least three sessions), minimum 8 hours for each session, and competency-based skill assessments; and periodic capacity-building training for continuous development.

***Compensation model***

Mean annual compensation for a heath manager: ¥70 000.^a^

Funding mechanism: Surplus of capitation allocated to WeDoctor.

Standard deployment: 1 full-time equivalent manager per primary health-care facility.

^a^ 1 United States dollar = ¥6.38.

## Approach

To restructure primary health care, Tianjin’s government partnered with WeDoctor in 2020 in a three-party agreement between the Health Commission, Insurance Bureau and WeDoctor. The Commission delegated control of diabetes management in community health centres to WeDoctor, including digital systems, service allocation and staffing. The Insurance Bureau introduced claims auditing by WeDoctor and capitation payment for enrolled outpatients with diabetes. Under this model, the Insurance Bureau set an annual expenditure ceiling for each patient’s outpatient diabetes care (US$ 1537 per enrolee, based on 2019 costs), capping payments to the provider (WeDoctor) rather than to patient benefits. WeDoctor kept savings generated by improving care efficiency and reducing unnecessary usage within this budget. By December 2022, WeDoctor had signed agreements with all 266 health centres throughout Tianjin, built operational capacity and enrolled patients. The pilot of patients enrolled under WeDoctor launched in January 2023.

### Capitation payment

Patients with diabetes voluntarily enrolled in the pilot which allowed choice between community health centres and hospitals. Patients who did not enrol continued to receive the pre-pilot usual care. The pilot offered benefits beyond the usual package including online consultations, the inclusion of complication-related medications in the insurance reimbursement scope, reduced co-payments and higher reimbursement ceilings.

The Insurance Bureau funding (about US$ 1537/person annually) covered all reimbursable services. Under the capitation budget, 30% of the generated surpluses were allocated to WeDoctor to recover investments (e.g. in health managers, AI, systems and equipment), and 70% went directly to the health centres. Hospitals kept 100% of their surpluses. 

### Third-party governance

During 2020–2024, WeDoctor raised about US$ 88 million through parent company capital and external financing. Revenue came from capitation-based shared savings and value-added preventive services.

#### Improving management

WeDoctor launched an initiative to strengthen governance capabilities across the community health centres. The company established an integrated health information platform that centralized financial management systems for all contracted health centres, incorporating AI-powered modules designed to audit medical prescriptions. To optimize interdepartmental coordination, WeDoctor deployed 90 liaison managers to support the health centres. These managers implemented system training, conducted operational analytics and arranged monthly financial risk meetings to review fund use for each centre. When deficits occurred (i.e. expenditure for enrolled patients exceeded the capitation budget), managers worked with local teams to develop corrective measures.

#### Redesigning services

WeDoctor redesigned diabetes care services by deploying more than 200 health managers, who were trained by WeDoctor (Box 1). WeDoctor also (i) equipped all health centres with ophthalmological and podiatric screening devices; (ii) implemented AI-generated patient health risk stratification to create personalized care plans; and (iii) integrated medication management with insurance processing via a commercial digital platform connected to pharmaceutical logistics for direct-to-patient medication delivery.

The WeDoctor system was integrated with health centres’ electronic infrastructure through a mobile application, which formed an AI-powered health management platform developed by Zhejiang University’s Ruiyi Artificial Intelligence Research Centre. Using patient data from three leading diabetes care hospital networks, the platform used an algorithm to deliver targeted features (Box 2).

Box 2 **Functions provided by the WeDoctor’s AI-enabled platform for diabetes management, Tianjin, China**

The health-care management platform uses a general-purpose large language model, fine-tuned using human feedback to improve output quality, to generate diagnostic hypotheses from structured clinical data, such as chief complaints, medical history and vital signs, entered by general practitioners. The platform provides tiered recommendations:

- diagnostic guidance: differential diagnosis suggestions with probability weighting;
- investigative protocols: prioritized lists of required examinations, such as cardiac ultrasound and liver function panels; and
- referral triggers: automated identification of cases requiring specialist intervention based on algorithmic analysis of treatment response trajectories.

***Standardized screening infrastructure***

Operationalized across 89.5% (238/266) of community health centres, the WeDoctor deploys uniform retinal imaging equipment; AI-assisted image interpretation validated against radiologist benchmarks; and centralized verification workflow for abnormal findings through a central teleradiology verification team.

***AI-enhanced prescription optimization***

The AI system analyses the patient’s data, identifies similar cases from its own database and recommends treatment options. These suggestions consider the medication history, adverse drug reactions, medication inventory, formulary availability and price. Treatment options are ranked by total cost for possible more cost-effective choices. Once a general practitioner submits a prescription, the AI system reviews it based on clinical guidelines, hospital-level data and insurance policies. The review results are categorized into three outcomes: flagged for revision, forwarded to a pharmacist for further review or approved directly.

***AI-enhanced risk-stratified patient follow-up system***

The health-care management platform operates through a five-component framework:

- data collection: health managers systematically input patient data at initial enrolment and each subsequent follow-up consultation (follow-up assessment with 76 indicators);
- automated risk classification: the system performs multiparametric evaluation using HbA1c levels (the main biomarker) and supplementary biomarkers to classify patients into three risk tiers;
- tiered management approach: the AI-enabled system classifies patients into three risk tiers with corresponding follow-up reminders: red (about 8%) with monthly reminders; yellow (24%) with reminders every 2 months; and green (68%) with quarterly reminders.
- personalized care delivery: the platform generates individualized plans incorporating evidence-based pharmacological regimens, tailored nutritional protocols, customized exercise prescriptions and lifestyle modification strategies.
- clinical support features: health-care supervisors receive automated monitoring alerts and scheduled complication screening protocols.

AI: artificial intelligence; HbA1c: glycated haemoglobin.

The health managers undertook care coordination through WeDoctor’s platform. Core responsibilities included biometric data collection (blood pressure readings and photograph-verified diet and exercise documentation); standardized screenings for diabetic complications; quarterly refinement of AI-generated health plans; and implementation of automated follow-up alerts with health education delivery via instant messaging and telephone and face-to-face consultation.

## Relevant changes

From June to December 2022, 494 945 patients with diabetes enrolled in one of three models: 18.2% (90 064/494 945) in WeDoctor community health centres; 27.2% (134 461/494 945) in hospital-based care; and 56.5% (279 423/494 945) in usual care. By December 2023, overall retention was 86.2% (426 876/494 945). Attrition rates varied across groups: 17.1% (15 375/90 064) for WeDoctor health centres; 22.5% (30 285/134 461) for hospital-based care; and 11.2% (31 412/279 423) for usual care. Usual care had the lowest attrition rate, likely reflecting differences in patient characteristics and payment incentives rather than a deficiency in the WeDoctor model. Additionally, WeDoctor’s attrition rate was lower than for hospital-based care, indicating its relative effectiveness in patient retention under a managed budget.

In 2023, the WeDoctor model demonstrated financial viability. Against capitation funds of US$ 152.04 million, expenditure was US$ 114.42 million, generating a US$ 37.62 million surplus shared between health centres and WeDoctor. Concurrently, all participating health centres saw a 65.0% (US$ 154 577/237 805) revenue growth during 2022–2023 for diabetes care. Physicians’ annual salaries rose by 30.0% (US$ 5172/17 241), correlating with high reported levels of satisfaction with and trust in physicians’ work (78.9%; 258/327) and competence (71.9%; 235/327).

The WeDoctor model shifted care to health centres, which increased their share of outpatient visits and diabetes-related costs, while hospitals saw a decline. Conversely, inpatient spending rose in all groups. As shown in Table 1, the WeDoctor health centre group had a 2.6% (0.7/26.6) increase in diabetes-related outpatient visits between 2022 and 2023, contrasting with declines of 10.6% (−3.8/35.7) and 2.3% (−0.8/34.1) in the hospital-based and usual care groups, respectively. This pattern was not seen in non-diabetes care, where all groups showed similar increases. The WeDoctor group captured 69.2% (1 650 359/2 384 912) of all outpatient visits in 2023, a 7.1 percentage point increase from 2022, exceeding the 2–3 percentage point gains in the other groups. Inpatient admissions rose substantially in all groups with no significant differences, likely due to the health-care financing structure which emphasizes inpatient reimbursement.

**Table 1:** Health-care use and medical expenditure, China, 2022–2023

Variable	WeDoctor community health centre group (*n* = 74 689)					Hospital-based provider group (*n* = 104 176)					Usual care group (*n* = 248 011)			
	2022	2023	Absolute difference (95% CI)	Relative difference, %		2022	2023	Absolute difference (95% CI)	Relative difference, %		2022	2023	Absolute difference (95% CI)	Relative difference, %
**Care utilization**														
Average annual outpatient visits per patient	59.1	63.1	4.0 (3.8 to 4.2)	6.8		72.7	72.4	−0.4 (−0.6 to −0.1)	−0.5		71.2	75.2	4.0 (3.8 to 4.2)	5.6
Diabetes-related visits	26.6	27.3	0.7 (0.6 to 0.8)	2.6		35.7	31.9	−3.8 (−3.9 to −3.7)	−10.6		34.1	33.3	−0.8 (−0.9 to −0.7)	−2.3
Non-diabetes-related visits	32.4	35.7	3.3 (3.1 to 3.5)	10.2		37.0	40.5	3.4 (3.3 to 3.6)	9.3		37.1	41.8	4.8 (4.6 to 4.9)	12.9
% of outpatient visits at community health centres	58.0	61.5	3.5 (3.1 to 3.9)	6.0		56.1	57.6	1.5 (1.2 to 1.8)	2.7		59.7	61.8	2.1 (1.8 to 2.4)	3.5
Diabetes-related visits	62.2	69.2	7.1 (6.6 to 7.5)	11.4		57.3	59.0	1.7 (1.3 to 2.1)	3.0		65.5	68.6	3.1 (2.7 to 3.5)	4.7
Non-diabetes-related visits	54.6	55.7	1.0 (0.6 to 1.5)	1.9		54.9	56.5	1.6 (1.2 to 1.9)	2.8		54.4	56.5	2.0 (1.8 to 2.3)	3.7
Average annual hospital admissions per patient	0.4	0.5	0.1 (0.1 to 0.1)	25.8		0.4	0.4	0.1 (0.1 to 0.1)	27.5		0.4	0.5	0.1 (0.1 to 0.1)	26.2
**Medical expenditure**														
Annual outpatient expenditure per patient, in ¥**^a^**	11 274.5	10 858.0	−416.4 (−474.8 to −358.1)	−3.7		17 682.8	16 230.0	−1452.9 (−1511.3 to −1394.5)	−8.2		18 566.0	18 199.0	−366.9 (−428.7 to −305.2)	−2.0
Diabetes-related visits	6 134.1	5 339.9	−794.1 (−824.2 to −764.1)	−12.9		10 964.6	9 024.9	−1939.7 (−1975.6 to −1903.9)	−17.7		11 105.5	9 929.8	−1175.6 (−1210.2 to −1141.0)	−10.6
Non-diabetes-related visits	5 140.4	5 518.1	377.7 (327.5 to 427.9)	7.3		6 718.2	7 205.0	486.9 (441.1 to 532.6)	7.2		7 460.5	8 269.2	808.7 (760.5 to 856.8)	10.8
Community health centre share of total health-care expenditure, %	48.3	50.5	2.1 (1.7 to 2.6)	4.4		47.2	46.7	−0.5 (−0.8 to −0.2)	−1.3		57.8	62.6	4.7 (4.4 to 5.1)	8.2
Diabetes-related visits	54.3	60.2	5.8 (5.3 to 6.4)	10.7		48.1	45.3	−2.8 (−3.2 to −2.5)	−6.0		64.2	69.8	5.7 (5.3 to 6.1)	8.8
Non-diabetes-related visits	41.1	41.0	−0.1 (−0.6 to 0.5)	−0.1		45.8	48.5	2.7 (2.2 to 3.2)	5.0		48.4	53.8	5.4 (4.9 to 6.0)	11.2
Annual inpatient expenditure per patient, in ¥**^a^**	5 931.2	7 345.7	1 414.5 (1 219.0 to 1 610.1)	23.8		6 145.6	7 923.8	1 778.2 (1 597.5 to 1 958.8)	28.9		7 317.3	8 234.2	916.9 (782.1 to 1 051.7)	12.5

CI: confidence interval; ¥: yuan.

^a^ 1 United States dollar = ¥6.38.

Note: Inconsistencies arise in some values due to rounding.

All groups saw outpatient expenditure reductions: –3.7% (–416.4/11 274.5) for WeDoctor health centres; −8.2% (−1452.9/17 682.8) for hospital-based care; and −2.0% (−366.9/18 566.0) for usual care. These reductions were driven by decreases in diabetes-related claims (indicating improved patient health outcomes and more efficient resource utilization). 

## Lessons learnt

Tianjin’s chronic care pilot demonstrates that revitalizing primary care requires overcoming the dichotomy between vertical disease-specific programmes (often lacking cross-sectoral coordination) and horizontal system-strengthening efforts (often lacking disease-specific focus). Tianjin’s innovative so-called diagonal governance mechanism resolved this disconnect by using a private third-party as an intermediary: it used diabetes management as a vertical entry point to drive accountability, while simultaneously building horizontal capacity (AI and health managers) to strengthen the primary care system. This approach showed that: (i) capitation sharing can align insurer, provider and patient interests; (ii) AI decision support can help upgrade primary care capacity; and (iii) administrative constraints can be resolved. Box 3 summarizes the main lessons learnt. A stakeholder analysis that identified interests, risks and mitigation strategies is available in the online repository.^5^

Box 3 **Summary of main lessons learnt**

- The adoption of third-party governance and public–private partnerships significantly strengthened coordination between health workers and insurance systems, fostering more integrated and efficient service delivery.
- Expanding and enriching primary care capacity for chronic disease management increased the attractiveness and accessibility of public health services, effectively meeting community needs for integrated care.
- The combined implementation of capitation payments and prescription surveillance using artificial intelligence reduced inappropriate medication use, decreased unnecessary by-passing of primary health care, and contained insurance expenditures, thus ensuring more sustainable resource allocation.

Tianjin’s model represents a shift from conventional public–private partnerships, which act as a stopgap for acute resource deficits.^6^^,^^7^ The new model shows that using an existing community health centre network can drive systemic upgrading. Furthermore, private-sector governance and AI implementation can simultaneously improve care quality and integrate insurers with providers. 

The model also shows that evidence-based principles from high-income countries (sustained financing, individualized care and decision support) can be translated into a public–private partnership framework in a middle-income country. Embedding private-sector efficiencies within a shared savings mechanism can improve population health and cost containment without additional public expenditure, thereby offering solutions that could be transferred to health systems worldwide.

After the initial success, Tianjin expanded the model in 2024 to cover 13 chronic conditions. Interest has spread to eight provinces, although scale-up faces structural barriers including payment misalignment, distrust of private partners, patient disenrollment, fiscal constraints and hospital resistance. Successful replication requires municipal commitment, capable private partners, primary care engagement, patient retention and adequate financing, underscoring the need for robust regulation to prevent profit-driven quality compromise.
